# Occurrence of bipolar spectrum disorder and comorbidities in women with eating disorders

**DOI:** 10.1186/2194-7511-1-25

**Published:** 2013-11-13

**Authors:** Rodolfo Nunes Campos, Domingos Junior Rodrigues dos Santos, Táki Athanássios Cordás, Jules Angst, Ricardo Alberto Moreno

**Affiliations:** Mood Disorders Unit (GRUDA), Department and Institute of Psychiatry, School of Medicine, University of São Paulo (HC-FMUSP), Rua Ovidio Pires de Campos 785, São Paulo, 05403-010 Brazil; Department of Psychiatry, Federal University of Goias, Goias, 74690-900 Brazil; Eating Disorders Unit (AMBULIM), Department and Institute of Psychiatry, School of Medicine, University of São Paulo (HC-FMUSP), São Paulo, 05403-010 Brazil; Psychiatric Hospital, Zurich University, Zurich, 8091 Switzerland

**Keywords:** Bipolar disorder, Bipolar spectrum, Eating disorders, Comorbidity

## Abstract

**Background:**

Eating disorder (ED) patients often have comorbidities with other psychiatric disorders, especially with mood disorders. Although recent studies suggest an intimate relationship between ED and bipolar disorder (BD), the study on a broader bipolar spectrum definition has not been done in this population. We aimed to study the occurrence of bipolar spectrum (BS) and comorbidities in eating disorder patients of a tertiary service provider.

**Methods:**

Sixty-nine female patients diagnosed with anorexia nervosa, bulimia nervosa, or eating disorder not otherwise specified were evaluated. The assessment comprised the Structured Clinical Interview for DSM-IV Axis I Disorders (SCID-I), clinical criteria for diagnosis of the Zurich bipolar spectrum. Mann–Whitney tests compared means of continuous variables. The association between categorical variables and the groups was described using contingency tables and analyzed using the chi-square or Fisher's exact test. The level of significance alpha was set at 5%.

**Results:**

The results showed that 68.1% of patients had comorbidity with bipolar spectrum, and this was associated with higher family income, proportion of married people, and comorbidity with substance use. The ED with BS group showed higher rates of substance use comorbidity (40.4%) than the ED without BS group (13.6%).

**Discussion:**

These results showed that the bipolar spectrum is a common comorbidity in patients with eating disorders and is associated with correlates of clinical importance, notably the comorbidity with substance use. Due to the pattern of similarity between the groups with and without comorbid bipolar spectrum in relation to various outcomes evaluated, the identification of comorbidity can be difficult. However, the precise diagnosis and careful identification of clinical correlates may contribute to future advances in treating these conditions. Further studies are necessary to evaluate the association of other clinical correlates and its possible causal association.

## Background

There is a growing body of evidence concerning bipolar disorder (BD) comorbidity in patients with eating disorder (ED) (Blinder et al. 2006;McElroy et al. 2005). Higher rates of BD compared to the general population have been reported among ED patients (Hudson et al. 2007;McElroy et al. 2005) and have also been associated with early onset, suicidal ideation, residual symptoms, treatment resistance, and worsening course (Wildes et al. 2007;McElroy et al. 2001;Fedorowicz et al. 2007;Kaye et al. 2004).

The wide range in rates of BD comorbidity in patients with ED is likely due to the use of different definitions of bipolarity and the boundaries between ED and BD (McElroy et al. 2005). Halmi et al. (1991) found higher rates of atypical BD in female anorexia nervosa (AN) patients than in the control group (13.3% vs. 0%). Evaluating a community sample, Hudson et al. (2007) found the occurrence of bipolar I (BP I) and II (BP II) disorders ranging from 3% in AN to 17.7% in bulimia nervosa (BN), including 12.5% in binge eating disorder (BED) and 10.5% in subthreshold BED.

Moreover, the prevalence of EDs not meeting the formal criteria for AN or BN, such as ‘eating disorders not otherwise specified’ (EDNOS) or subthreshold ED spectrum disorders is considerably higher in BD patients (up to 3.6% to 10%) (McElroy et al. 2005). Evaluating 50 AN patients regarding manic and depressive symptoms, Wildes et al. (2007) found that 86% presented threshold symptoms for depression and 60% for mania, and it was associated with a history of self-induced vomiting and suicidality.

Despite the recognized association between mood disorders and ED, few studies described the relationship with BD and its broader definition of bipolar spectrum (BS). Most studies have been conducted using the DSM-III-R criteria, and the estimates of prevalence of comorbid disorders in psychiatric settings may be skewed by the imperfect recognition of BD (Mantere et al. 2004).

To our knowledge, the prevalence of soft forms of BD (i.e., bipolar spectrum) in ED patients is not yet reported. There is a need to analyze the prevalence and correlates of BS in ED, first because BS may be underdiagnosed due to phenomenology overlap and the severity of ED. Second, the axis I comorbidity pattern could be clinically useful in helping recognize BS in this population. Third, understanding this overlap raises the question of shared vulnerability and, possible, physiopathology. The aim of this study was to determine the prevalence of BS in a well-defined sample of ED patients, evaluating the prevalence of other axis I disorders.

## Methods

The methods of ESPECTRA project are described in greater details elsewhere (Campos et al. 2011). Women aged 18 to 45 years were recruited from the Eating Disorder Unit (AMBULIM) of the Institute of Psychiatry of the Clinical Hospital of the University of São Paulo, School of Medicine. Considering that ED is much more frequent in women than in men (Woodside et al. 2001), we just enrolled females in order to exclude a possible gender effect. Patients were included if they met the DSM-IV-TR criteria for AN, BN, or EDNOS. BED patients were excluded, and the absence of other comorbidities is not an exclusion criterion (Campos et al. 2011).

The evaluation included completion of the Structured Clinical Interview for DSM-IV Axis I Disorders (SCID-I) (First et al. 1996) to establish the diagnosis of eating disorders and comorbidities (psychotic disorders, mood disorders, anxiety disorders, alcohol and substance use disorders). Substances evaluated were amphetamine, cannabis, cocaine, hallucinogen, opioids, and sedative/hypnotic/anxiolytic. A clinical interview based on the Zurich criteria identified the BS, and structured questionnaires determined demographic factors, illness information, and family history.

Details of the definition of BS are described elsewhere (Angst et al. 2003). According to the redefinition of hypomania based on the concept of overactivity, the Zurich criteria consider a hypomanic episode duration of at least 1 day and no exclusion criteria, such as hypomania induced by an antidepressant, substances (stimulants, alcohol, etc.), and other causes (corticosteroids, brain lesion). Patients were divided into BS subtypes: BP I, BP II, minor bipolar disorder (MinBP), and pure hypomania (Pure Hyp).

A. Hypomanic syndrome
  Episode of at least two hypomanic symptoms without consequences
  Euphoria, irritability, or overactivity
  Have personally experienced problems or received comments from others that something must be wrong with them (consequences)
B. Hypomanic symptoms
  Present at least three out of seven signs and symptoms of DSM-IV hypomania

The bipolar spectrum is defined as follows:

1. BD I: major depressive episodes associated with mania
2. BD II: major depressive episodes associated with (a) hypomanic syndrome or (b) hypomanic symptoms
3. BD *minor*: dysthymia, minor depression, or recurrent brief depression associated with (a) hypomanic syndrome or (b) hypomanic symptoms
4. Pure hypomania: hypomanic syndrome (a) without diagnosis of depression and (b) hypomanic symptoms only

The protocol was approved by the local ethics committee, and it conformed to the 2008 Helsinki Declaration (CAPPesq protocol number: 1154/09). Written informed consent was obtained from all participants prior to any study-related activities.

Data were analyzed using SPSS for Windows (version 14.0, SPSS, Chicago, IL, USA). Descriptive statistics included means and standard deviations (SD) for continuous variables and percents for categorical variables. Chi-square test and student's *t* test or Mann–Whitney were used for group comparisons. All statistical tests were two-tailed, and a significant level of 5% was considered for all tests.

## Results

A total of 69 individuals with ED were evaluated. The mean current age was 27.9 years (SD 6.4), most of the participants were not married (72.5%), and the mean offspring number was 0.46 (SD 0.98). Patients reported 13.3 years (SD 3.5) of education, 63.8% were not working, and 36.2% had no religion.

Most patients had comorbidity with BS (68.1%), and it was associated with higher income (Table 1). Patients with BS comorbidity were more likely to be married and have more children than patients without BS. There was no difference regarding mean age, level of education, number of family members with other psychiatric disorders, reported ethnicity, and religion.

**Table 1:** **Demographic and clinical features of ED patients with and without BS comorbidity**

Variable		ED without BS (***N*** = 22)		ED with BS (***N*** = 47)		***P*** value
		Mean (SD)	CI (95%)	Mean (SD)	CI (95%)	
Age (years)		27.59 (5.09)	25.34-29.85	27.98 (7.04)	25.91-30.05	0.995^a^
Offspring		0.09 (0.29)	0.00-0.22	0.64 (1.13)	0.31-0.97	0.030^a^
Level of education (years)		12.64 (4.17)	10.79-14.49	13.61 (3.13)	12.69-14.52	0.126^a^
Family income (R$)^c^		3,102.38 (4,196.26)	1,192.27-5,012.50	3,597.34-(2,497.63)	2,864.01-4,330.67	0.031^a^
First-degree relative with BD		0.05 (0.21)	0.00-0.14	0.11 (0.37)	0.00-0.22	0.547^a^
First-degree relative with depression		0.50 (0.74)	0.17-0.83	0.45 (0.54)	0.29-0.61	0.929^a^
First-degree relative with other mental disorders		0.09 (0.29)	0.00-0.22	0.21 (0.59)	0.04-0.39	0.481^a^
Relative with BD		0.09 (0.29)	0.00-0.22	0.13 (0.40)	0.01-0.24	0.825^a^
Relative with depression		0.91 (1.44)	0.27-1.55	1.17 (1.32)	0.78-1.56	0.171^a^
Relative with other mental disorders		0.32 (0.57)	0.07-0.57	0.38 (0.64)	0.19-0.57	0.712^a^
		*N*	%	*N*	%	
Married	No	20	90.9	30	63.8	0.019^b^
	Yes	2	9.1	17	36.2	
Ethnicity	Caucasian	7	31.8	28	59.6	0.186^b^
	Black	1	4.5	2	4.3	
	Asia	1	4.5	1	2.1	
	Other	13	59.1	16	34.0	
Occupation	Working	7	31.8	18	38.3	0.511^b^
	Retired	3	13.6	7	14.9	
	Never worked	4	18.2	3	6.4	
	Unemployed	8	36.4	19	40.4	

ED, eating disorder; BS, bipolar spectrum; CI, confidence interval. ^a^Mann-Whitney; ^b^chi-square or Fisher's exact test; ^c^Brazilian reais.

High rates of comorbidities were found in both groups of ED with and without BS, but there was no difference in rates of any specific anxiety disorder (Table 2). The ED with BS group showed higher rates of substance use comorbidity (40.4%) than the ED without BS group (13.6%) (OR = 4.298; *P* = 0.03).

**Table 2:** **Axis I comorbidities in ED with and without BS**

	ED without BS (***N*** = 22)		ED with BS (***N*** = 47)		***P*** value	OR	CI (95%)	
	***N***	%	***N***	%			Lower limit	Upper limit
Major depressive disorder	20	90.9	21	44.7	0.000	0.164	0.043	0.631
Dysthymic disorder	1	4.5	-	0.0	0.319	0.955	0.871	1.046
Bipolar I disorder	-	0.0	18	38.3	0.000	1.621	1.294	2.030
Bipolar II disorder	-	0.0	6	12.8	0.090	1.146	1.028	1.279
Cyclothymic disorder	-	0.0	-	0.0		NA^a^		
Alcohol abuse	2	9.1	5	10.6	1.000	1.190	0.212	6.676
Alcohol dependence	3	13.6	8	17.0	1.000	1.299	0.309	5.460
Alcohol use^b^	5	22.7	13	27.7	0.774	1.300	0.398	4.249
Substance abuse	-	0.0	2	4.3	0.461	1.044	0.983	1.109
Substance dependence	3	13.6	17	36.2	0.086	3.589	0.926	13.917
Substance use^c^	3	13.6	19	40.4	0.03	4.298	1.114	16.575
Alcohol/substance use^d^	7	31.8	22	46.8	0.300	1.886	0.650	5.467
Panic disorder	8	36.4	19	40.4	0.797	1.187	0.417	3.380
Agoraphobia without panic disorder	2	9.1	2	4.3	0.587	0.444	0.058	3.382
Social phobia	11	50.0	16	34.0	0.290	0.516	0.184	1.447
Specific phobia	4	18.2	10	21.3	1.000	1.216	0.335	4.414
Obsessive-compulsive disorder	5	22.7	12	25.5	1.000	1.166	0.353	3.845
Post-traumatic stress disorder	2	9.1	4	8.5	1.000	0.930	0.157	5.507
Generalized anxiety disorder	-	0.0	3	6.4	0.546	1.068	0.991	1.151

Fisher's exact test was used. ED, eating disorder; BS, bipolar spectrum; OR, odds ratio. ^a^OR not applicable due to the existence of cells with zero frequency; ^b^combination of alcohol abuse and dependence; ^c^combination of drug abuse and dependence; ^d^alcohol/substance use.

## Discussion

The present study shows a high prevalence (68.1%) of BS in a well-defined population of 69 ED patients. This high rate of BS is compatible with the findings of Alciati et al. (2007) of 89% in 83 severely obese patients seeking surgical treatment. The definition of BS has been supported by many studies showing that it is a highly prevalent condition with clinical implications, and there is a need to redefine comorbidities according to this knowledge (Akiskal et al. 2000). The Zurich definition of BS (Angst et al. 2003), epidemiology based, enriched the understanding of soft bipolarity challenging the current DSM concepts. The broader definitions result directly in strong increase of the prevalence of BS. A reanalysis of the database of the Epidemiological Catchment Area (ECA) study indicated that 6.4% of the general population met the criteria for bipolar spectrum (Judd and Akiskal 2003), while the original study found 0.8% for BD. Data from the Sao Paulo Epidemiological Catchment Area study showed that the lifetime prevalence of BS was 8.3% (the softer forms of BD representing 6.6%) and it was of great clinical importance (Moreno and Andrade 2005;Moreno and Andrade 2010). Merikangas et al. (2011) in a large study across 11 countries found that the overall prevalence of BS was 2.4%. In addition to the findings mentioned above, we extended previous investigations by demonstrating that ED is associated with BS in its broader definition.

The BS is underdiagnosed in clinical practice (Angst et al. 2003;Bschor et al. 2012), and comorbidity often is an important factor that contributes to the delay in its identification. Patterns of psychiatric comorbidity suggest one possible strategy for improving recognition of bipolar disorder among patients presenting with depressive symptoms, and these patients may benefit from additional screening for bipolar disorder (Matza et al. 2005). We compared the overall comorbidity profile of ED patients with or without bipolar spectrum to obtain a comprehensive view of the differences of current comorbidity with ED. We found patterns of psychiatric comorbidity in ED with and without BS to differ somewhat qualitatively. This finding is in line with the results of previous studies that indicate an association of ED with greater substance use (Bulik et al. 2004).

Because of the severity of ED, comorbidities with other axis I disorders may be neglected. It is important to note that anxiety disorders and substance use are also highly prevalent, representing conditions that should be systematically assessed. The understanding of how comorbidities can modify the course and outcome in this population can be of clinical utility both in the diagnostic and treatment field. Once these comorbidities become more frequent than would be expected, it raises the question about pathophysiological implications.

These findings must be considered in view of some methodological limitations. First, historical illness variables were obtained retrospectively. Second, our group of ED patients was selected in a tertiary and very specialized research center and may not be representative of the community, and results may not be generalized. Our study has several strengths such as the use of specific and validated criteria for a broader definition of BD, and structured interview and validated instruments for the evaluation of axis I comorbidities.

## Conclusions

This study supports previous data (McElroy et al. 2005;Brietzke et al. 2011;Hudson et al. 2007) concerning the relationship of ED and bipolar disorders, and shows the importance of carefully evaluating ED patients. BS is highly prevalent in women with ED and strongly associated with substance use disorder comorbidity. A common liability may underlie the expression of these disorders in a single externalizing spectrum. One possibility is that this liability is continuous and the risk of externalizing disorders is graded. Another possibility is that the liability underlying the externalization of spectrum disorders is categorical such that individuals fall into groups of liability (Markon and Krueger 2005). A broader evaluation of psychiatric disorders beyond the standardized categorization criteria can contribute to a more realistic understanding of comorbidities.

A much more comprehensive assessment and measurement of psychopathology can enhance operational diagnoses and lead clinicians to broader examinations (Angst 2007), and lifetime substance use is a correlate that can be of great importance in differentiating disorders that have common characteristics. This kind of information can build a body of evidence supporting a dimensional approach in diagnosing these conditions, and as long as new studies incorporate this wider approach, the results will be directly seen in the number of patients diagnosed.

## Abbreviations

AMBULIM: Eating Disorder Unit
AN: Anorexia nervosa
BD: Bipolar disorder
BED: Binge eating disorder
BN: Bulimia nervosa
BP I: Bipolar I disorder
BP II: Bipolar II disorder
BS: Bipolar spectrum
DSM-III-R: Diagnostic and Statistical Manual of Mental Disorders, third edition, revised
DSM-IV-TR: Diagnostic and Statistical Manual of Mental Disorders, fourth edition, text revision
ECA: Epidemiological Catchment Area
ED: Eating disorder
EDNOS: Eating disorder not otherwise specified
ESPECTRA: Occurrence of bipolar spectrum disorders in eating disorder patients
MinBP: Minor bipolar disorder
Pure Hyp: Pure hypomania
SCID-I: Structured Clinical Interview for DSM-IV Axis I Disorders
SPSS: Statistical Package for the Social Sciences
